# Supplementary material for: Ca2+-Dependent and -Independent Calmodulin Binding to the Cytoplasmic Loop of Gap Junction Connexins
Source: Int J Mol Sci. 2023 Feb 19;24(4):4153. doi: 10.3390/ijms24044153 (PMC9961272; doi:10.3390/ijms24044153)
Supplement: Supplementary file 1 [file ijms-24-04153-s001.zip › ijms-2217003-supplementary.pdf]

International Journal of
Molecular Sciences

MDPI

Supplementary Material

Supplementary Methods

Analysis of cell number. To determine the number of cells per field of view we counted the number of Hoechst stained nuclei using a simple macro (Nuclei count.ijm) recorded in Fiji ImageJ.

Counting cell nuclei using Fiji ImageJ. Briefly, images were background subtracted with a rolling ball method before images were smoothed and thresh-holed. Images were then converted to a mask, a Watershed applied to identify and separate overlapping nuclei and objects counted using the Analyze particles function. Data was then imported into GraphPad prism for analysis.

Analysis of cell viability. To determine if the gap junction inhibitors or CaM peptides used here caused cell death, we determined the number of PI+ cells per field (excluding the lesion area), using a simple macro (PI+count.ijm) recorded in Fiji ImageJ. Cell viability was expressed as the fraction of cells negative for PI.

Analysing cell viability: Counting numbers of PI+ cells. Briefly, images were background subtracted with a rolling ball method before images were smoothed and thresh-holed. Images were then converted to a mask, and a rectangle placed over the lesion region. The contents of the rectangle were removed and PI+ objects counted using the Analyze particles function. Data were then imported into GraphPad prism for analysis.

Supplementary Data

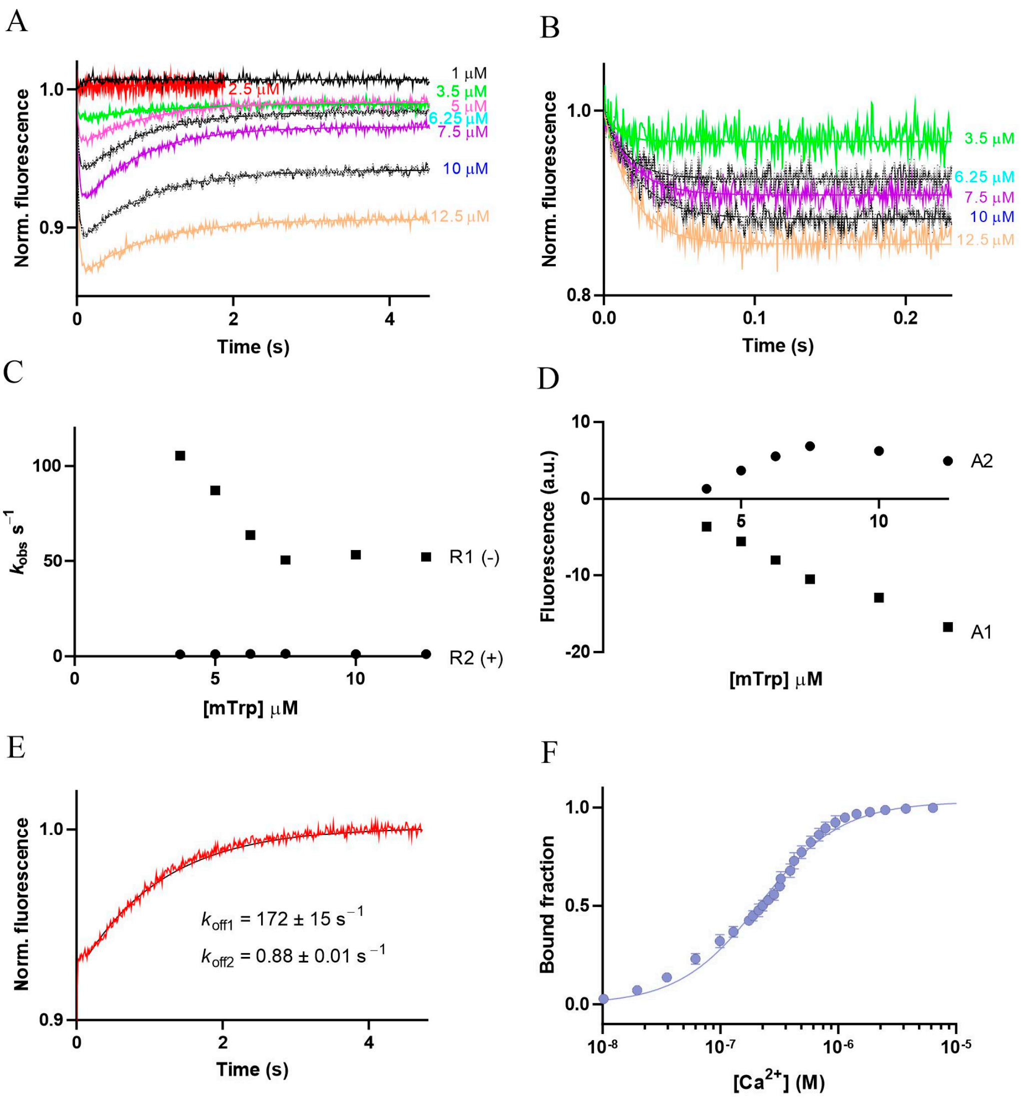

Supplementary Figure S1. Kinetics of Ca^{2+}- independent interactions of mTrp peptide with DA-CaM and Ca^{2+} dependence of interaction measured by TA-CaM. (**A**) On association of mTrp and apo-DA-CaM, there is a clear, biphasic fluorescence change: initial quenching is followed by a second isomerisation in which DA-CaM partially stretches back. (**B**) The initial rapid phase of binding is shown on an extended timescale. Plot of (**C**) the observed rates of association and (**D**) the amplitudes of decreasing phase one and increasing phase two. (**E**) mTrp dissociation from apo-DA-CaM occurs by a fast, 130 s^{-1} and a slow, 0.9 s^{-1} phase, in both the fluorescence increases, indicating that the reverse pathway is different from the on-pathway. Rapid dissociation of the apo-DA-CaM complex is consistent with specific but lower affinity interaction, compared to Cx CL2 peptides, as expected for a peptide derived from smooth muscle (sm) MLCK, an enzyme highly Ca^{2+}-dependently regulated by CaM. (**F**) Ca^{2+} dependence of TA-CaM fluorescence in the presence of mTrp peptide (Table 2). Significant binding at 50–100 nM [Ca^{2+}] is consistent with previously proposed C-lobe anchoring of CaM to sm MLCK in resting cells (56).
